# Association between dyslipidemia and vitamin D deficiency: a cross-sectional study in Chinese healthy population

**DOI:** 10.3389/fendo.2025.1450924

**Published:** 2025-04-30

**Authors:** Bin Li, Jianhong Wang, Jialie Xu, Jianying Xie, Quanyong Liu, Chenxi Yang, Zhengmao Zhang

**Affiliations:** Department of orthopaedics, Yuhuan people’s hospital, Taizhou, Zhejiang, China

**Keywords:** 25-hydroxyvitamin D, Dyslipidemia, vitamin D Deficiency, cholesterol, triglycerides

## Abstract

**Background:**

Given the global prevalence of vitamin D deficiency, this study examines the association between serum 25-hydroxyvitamin D (25(OH)D) levels and lipid profiles, including total cholesterol, low-density lipoprotein cholesterol (LDL-C), high-density lipoprotein cholesterol (HDL-C), and triglycerides (TG).

**Methods:**

In a cross-sectional analysis at the Yuhuan People’s Hospital Physical Examination Center, 1535 asymptomatic individuals underwent anthropometric assessment and blood sampling. Serum 25(OH)D levels were quantified by a chemiluminescent immunoassay, while lipid parameters were evaluated by an enzymatic method on a fully automated analyzer.

**Results:**

The mean serum 25(OH)D level of the participants was 49.6 ± 15.3 nmol/L, with 54.7% demonstrating vitamin D deficiency. Female participants had significantly lower mean 25(OH)D levels (46.2 ± 13.7 nmol/L) than males (54.1 ± 16.0 nmol/L, P < 0.001). After adjustment for age and BMI, multivariate ordinal logistic analysis revealed a 1.71-fold higher dyslipidemia-associated risk of vitamin D deficiency in the population (OR=1.71, 95% CI=1.07-2.79), more pronounced in females(OR=2.84, 95% CI=1.17-8.09) compared to males (OR=1.44, 95% CI=0.82-2.58). Notably, vitamin D deficiency was significantly associated with elevated LDL-C levels in females (OR=5.17, 95% CI=1.47-32.96), in contrast to males.

**Conclusion:**

This study highlights a significant association between 25(OH)D deficiency and an increased risk of dyslipidemia, especially in females, highlighting the importance of considering gender-specific risk factors in the management of 25(OH)D deficiency.

## 1 Introduction

Vitamin D, a key fat-soluble nutrient, is critical not only for bone health (1), but also for its role in reducing insulin resistance (2, 3), modulating immune function (4), acting as an anti-inflammatory agent (5), and potentially reducing mortality rates (6). Despite its importance, vitamin D deficiency, which affects nearly one billion people worldwide, is a significant public health challenge (7). This deficiency varies widely across regions, from less than 20% in Northern Europe to over 80% in parts of the Middle East, highlighting the need for region-specific research (8–12).

Emerging evidence suggests an association between low 25(OH)D levels and dyslipidemia, a major contributor to the rising incidence of cardiovascular disease worldwide (13–15). Given the high prevalence of cardiovascular deaths in China, where dyslipidemia is alarmingly common, understanding this association in the Chinese population is particularly important (16).

While several epidemiologic studies have examined the association between dyslipidemia and 25(OH)D levels, they have predominantly focused on Western populations, with mixed results (17–21). This discrepancy underscores the urgent need for comprehensive studies in diverse demographic settings, particularly in China, where data remain scarce. The current study aims to fill this gap by investigating the relationship between lipid profiles and standardized 25(OH)D levels in healthy Chinese adults, providing insights into potential cardiovascular disease prevention strategies in this population.

Vitamin D may influence lipid metabolism through several mechanisms. First, it regulates the expression of genes involved in cholesterol synthesis and clearance, such as CYP7A1 and ABCA1 (22, 23). Second, vitamin D has been shown to modulate inflammatory pathways, which play a key role in the development of dyslipidemia (24, 25). Third, vitamin D may affect insulin sensitivity, which in turn influences lipid metabolism (26, 27). These mechanisms are supported by previous studies demonstrating that vitamin D supplementation can improve lipid profiles, particularly by reducing LDL-C and triglyceride levels (28, 29). Given these potential mechanisms, our study aims to further elucidate the relationship between vitamin D deficiency and dyslipidemia in a Chinese population, providing insights into the role of vitamin D in lipid metabolism and its implications for cardiovascular health.

## 2 Materials and methods

### 2.1 Study population

Our study population consisted of individuals undergoing medical examinations at Yuhuan People’s Hospital. We included adults over 18 years of age with complete data on serum 25(OH)D and lipid profiles. We excluded individuals with acute diseases, a history of cancer, chronic cardiac, pulmonary, hepatic, or renal diseases. Additionally, participants taking medications known to affect lipid metabolism or vitamin D levels, such as corticosteroids, androgens, estrogens, antipsychotics, anticonvulsants, and lipid-lowering agents, were excluded from the study.Using these criteria, 1,535 participants were included in the study.

The sample size was calculated using PASS software (version 2021) based on an expected effect size of 0.3, a significance level of 0.05, and a statistical power of 80%. Assuming a two-tailed test for the association between vitamin D deficiency and dyslipidemia, the calculation indicated that a minimum sample size of 1,200 participants was required to detect meaningful differences in 25-hydroxyvitamin D levels between groups with and without dyslipidemia. This calculation was informed by previous studies reporting an effect size of 0.3 for similar associations (17, 30). Although the calculated sample size was 1,200, we included 1,535 participants in the study to account for potential data variability and ensure robust statistical analysis.

### 2.2 Data collection

#### 2.2.1 Data collection

During the medical examination, we collected demographic and medical history data, including name, gender, age, and previous medical conditions. Participants’ weight and height were measured to the nearest 0.1 kg and 0.1 cm, respectively, without shoes or outer clothing. Body mass index (BMI) was calculated using the formula: weight in kg/(height in m)^2^.

#### 2.2.2 Laboratory measurements

Blood samples were collected from the antecubital vein after an overnight fast and centrifuged within 1 hour of collection. Total cholesterol, triglycerides, HDL-C, and LDL-C were quantified using a Beckman Coulter Chemistry Analyzer AU5800. Serum 25(OH)D levels were measured by chemiluminescence immunoassay (Mindray CL-8000i). We used the Endocrine Society guidelines to classify 25(OH)D status: deficiency (<50 nmol/L), insufficiency (50-75 nmol/L), and sufficiency (≥75 nmol/L) (30). Dyslipidemia was identified based on history of lipid-lowering treatment, clinical diagnosis, or lipid levels outside the reported normal ranges (31).

#### 2.2.3 Covariates

We identified sex, age, and BMI as potential confounders. BMI categories were defined as: normal weight (BMI 18.5-24.9), and overweight (BMI ≥25). Age subgroups were defined based on common classifications used in previous studies, with 40 years serving as a cutoff to distinguish between younger and middle-aged adults.

### 2.3 Statistical analysis

Data analysis was performed using R software, version 4.3.0. A p value of less than 0.05 was considered statistically significant. Continuous variables are presented as mean ± standard deviation, and categorical variables are presented as frequencies and percentages. Normality of continuous variables was assessed using the Shapiro-Wilk test. Variables that followed a normal distribution were presented as mean ± standard deviation, while non-normally distributed variables were presented as median (interquartile range). For normally distributed variables, parametric tests (Student’s t-test) were used; for non-normally distributed variables, non-parametric tests (Mann-Whitney U test) were applied. Univariate and multivariate ordinal logistic regression models adjusted for confounders were used to calculate odds ratios (ORs) and 95% confidence intervals (CIs) for the associations between the risk of 25(OH)D deficiency and dyslipidemia and lipid indicators.

#### 3 Results

The mean serum concentration of 25-hydroxyvitamin D (25(OH)D) among study participants was 49.6 ± 15.3 nmol/L. A remarkable 54.7% of the cohort was 25(OH)D deficient. Female participants had significantly lower mean 25(OH)D levels (46.2 ± 13.7 nmol/L) compared to their male counterparts (54.1 ± 16.0 nmol/L, P < 0.001). Consequently, a higher prevalence of 25(OH)D deficiency was observed in females(63.8%) than in males (42.8%, P < 0.001). Significant differences in age, BMI, and lipid profiles-including HDL, LDL, and triglycerides (TG)-were noted between the sexes (**Table 1**). Females had higher HDL levels (1.45 ± 0.31 mmol/L) compared with males (1.27 ± 0.25 mmol/L), whereas males had higher TG levels (1.77 ± 1.53 mmol/L vs. 1.13 ± 0.59 mmol/L).

**Table 1:** Mean (± SD) values for lipid panel in healthy participants.

	All (*N* = 1535)	Males (*n* = 664)	Females (*n* = 871)	*P* value
Age (in years)	45.95 (13.51)	46.85 (13.25)	45.26 (13.68)	0.022
Body mass index (kg/m^2^)	23.22 (3.30)	24.46 (3.12)	22.28 (3.12)	<0.001
25(OH)D (nmol/L)	49.6 (15.3)	54.1 (16.0)	46.2 (13.7)	<0.001
Total cholesterol (mmol/L)	5.05 (1.00)	5.08 (1.01)	5.03 (1.00)	0.421
Triglycerides (mmol/L)	1.41 (1.15)	1.77 (1.53)	1.13 (0.59)	<0.001
LDL-C (mmol/L)	3.32 (0.77)	3.37 (0.78)	3.27 (0.76)	0.010
HDL-C (mmol/L)	1.37 (0.30)	1.27 (0.25)	1.45 (0.31)	<0.001

**Table 2** shows the results of a univariate ordinal logistic analysis examining the association between 25(OH)D status, classified into three categories (<50 nmol/L, 50-75 nmol/L, and ≥75 nmol/L), and various health indicators, with the ≥75 nmol/L group serving as the reference. However, the small number of participants in this subgroup(25(OH)D levels ≥75 nmol/L) should be noted. This analysis revealed age-related differences in 25(OH)D levels, with younger participants having a higher prevalence of low 25(OH)D status compared to older individuals, a trend that was significant in both sexes. In addition, overweight males were more likely to be 25(OH)D deficient than their normal-weight counterparts, a relationship not observed in females.

**Table 2:** Univariate analysis showing unadjusted odds ratio and 95% CI between socio-demographic and lipid profile with low 25(OH)D in healthy participants.

Variables	Males (*N* = 664)				Females (*N* = 871)			
	25(OH)D ≤ 50 (*n* = 284)	50<25(OH)D<75 (*n* = 312)	25(OH)D≥75 (*n* = 68)	OR (95%CI)	25(OH)D ≤ 50 (*n* = 556)	50<25(OH)D<75 (*n* = 281)	25(OH)D≥75 (*n* = 34)	OR (95%CI)
Age in year categories								
Youth (<40y)	129 (45.42)	90 (28.85)	5 (7.35)	1	300 (53.96)	74 (26.33)	5 (14.71)	1
Middle-aged (≧40y,<65y)	148 (52.11)	189 (60.58)	38 (55.88)	0.20 (0.07, 0.48)	228 (41.01)	158 (56.23)	19 (55.88)	0.27 (0.09, 0.68)
Elderly (≧65y)	7 (2.46)	33 (10.58)	25 (36.76)	0.04 (0.01, 0.09)	28 (5.04)	49 (17.44)	10 (29.41)	0.10 (0.03, 0.30)
Body mass index (kg/m^2^)								
Normal	180 (63.38)	168 (53.85)	49 (72.06)	1	479 (86.15)	216 (76.87)	27 (79.41)	1
Overweight	104 (36.62)	144 (46.15)	19 (27.94)	1.84 (1.07, 3.27)	77 (13.85)	65 (23.13)	7 (20.59)	0.79 (0.35, 2.00)
Total cholesterol (mmol/L)								
Normal	235 (83.04)	278 (89.1)	64 (94.12)	1	488 (87.77)	241 (86.07)	31 (91.18)	1
High (≥6.22)	48 (16.96)	34 (10.9)	4 (5.88)	2.56 (1.02, 8.58)	68 (12.23)	39 (13.93)	3 (8.82)	1.52 (0.53, 6.39)
Triglycerides (mmol/L)								
Normal	211 (74.3)	260 (83.33)	61 (89.71)	1	530 (95.32)	258 (91.81)	34 (100)	1
High (≥2.26)	73 (25.7)	52 (16.67)	7 (10.29)	2.31 (1.10, 5.67)	26 (4.68)	23 (8.19)	0 (0)	4.99e+06 (3.67e-14, 2.83e+124)
LDL-C (mmol/L)								
Normal	232 (81.69)	270 (86.54)	62 (91.18)	1	488 (87.77)	235 (83.63)	32 (94.12)	1
High (≥4.14)	52 (18.31)	42 (13.46)	6 (8.82)	1.93 (0.88, 5.12)	68 (12.23)	46 (16.37)	2 (5.88)	2.52 (0.75, 15.70)
HDL-C (mmol/L)								
Normal	229 (80.63)	267 (85.58)	58 (85.29)	1	525 (94.42)	265 (94.31)	31 (91.18)	1
Low (<1.04)	55 (19.37)	45 (14.42)	10 (14.71)	1.17 (0.60, 2.50)	31 (5.58)	16 (5.69)	3 (8.82)	0.61 (0.21, 2.63)
Dyslipidemia								
No	141 (49.65)	195 (62.5)	46 (67.75)	1	437 (78.6)	200 (71.17)	28 (82.35)	1
Yes	143 (50.35)	117 (37.5)	22 (32.35)	1.62 (0.96, 2.80)	119 (21.4)	81 (28.83)	6 (17.65)	1.47 (0.64, 3.96)

A higher prevalence of 25(OH)D deficiency was observed in males with elevated total cholesterol (OR=2.56, 95% CI=1.02-8.58). This association was not significant in females (OR=1.52, 95% CI=0.53-6.39). Similarly, males with high triglyceride levels showed a trend toward 25(OH)D deficiency, an observation that was not replicated in females.

Multivariate ordinal logistic analysis adjusted for age and BMI showed that the risk of 25(OH)D deficiency associated with dyslipidemia was 1.71 times higher in the entire study population (OR=1.71, 95% CI=1.07-2.79). This risk was more pronounced in females (OR=2.84, 95% CI=1.17-8.09) than in males (OR=1.44, 95% CI=0.82-2.58) (**Table 3**). A significant association was found between 25(OH)D deficiency and high LDL-C in females (OR=5.17, 95% CI=1.47-32.96), which was not observed in males. No significant associations were found between vitamin D status and high total cholesterol or low LDL-C levels in either sex.

**Table 3:** Multivariate model showing the independent association of lipid panels with standardized 25-hydroxyvitamin D (25(OH)D) levels in males and females in healthy participants.

Variables	Total* OR_A_ (95%CI)	Males** OR_A_ (95%CI)	Females** OR_A_ (95%CI)
High total cholesterol (≥6.22 mmol/L)	2.35 (1.13, 5.73)	2.14 (0.83, 7.30)	2.92 (0.97, 12.74)
High triglycerides (≥2.26 mmol/L)	2.21 (1.05, 5.45)	1.73 (0.79, 4.36)	6.57e+06 (1.58e-12, 1.28e+117)
High LDL-C (≥4.14 mmol/L)	2.53 (1.26, 5.83)	1.79 (0.79, 4.86)	5.17 (1.47, 32.96)
Low HDL-C (<1.04 mmol/L)	1.00 (0.53, 2.01)	1.11 (0.54, 2.49)	0.73 (0.21, 3.47)
Dyslipidemia	1.71 (1.07, 2.79)	1.44 (0.82, 2.58)	2.84 (1.17, 8.09)

OR_A_ adjusted odds ratio.

*The model was adjusted for age, gender, body mass index.

**The model was adjusted for age, body mass index.

The extreme odds ratio (6.57e+06) and wide confidence interval (1.58e-12, 1.28e+117) for high triglycerides in females are likely due to the small sample size in this subgroup. This result should be interpreted with caution, as it may reflect data limitations rather than a true biological effect.

## 4 Discussion

Our study illuminates the intricate link between 25-hydroxyvitamin D [25(OH)D] deficiency and dyslipidemia. We discovered that individuals with 25(OH)D deficiency are markedly more likely to exhibit dyslipidemia, characterized by elevated total cholesterol and triglycerides. A significant association between 25(OH)D deficiency and elevated LDL-C levels in females (OR=5.17, 95% CI=1.47-32.96). This finding is consistent with previous studies suggesting that vitamin D may modulate lipid metabolism, potentially through its effects on gene expression related to cholesterol synthesis and clearance (32). The stronger association observed in females may be due to differences in fat distribution and hormonal influences on lipid metabolism (33), highlighting the need for gender-specific approaches to managing dyslipidemia and vitamin D deficiency. Contrary to some previous studies, we did not find a significant association between vitamin D status and HDL-C levels. This discrepancy may be due to differences in study populations, methodologies, or the influence of unmeasured confounding factors such as physical activity and dietary habits (34, 35). Future studies with larger sample sizes and longitudinal designs are needed to clarify this relationship.This finding contributes to the growing body of evidence on the significant health implications of 25(OH)D deficiency and dyslipidemia.

In **Table 3**, we observed an extremely high odds ratio (6.57e+06) for high triglycerides in females, with a wide confidence interval (1.58e-12, 1.28e+117). This extreme value is likely due to the small sample size in this subgroup, which may have led to unstable estimates. While this result should be interpreted with caution, it does not detract from the overall findings of our study. Future research with larger sample sizes is needed to further investigate this association.

Considering cross-sectional studies examining the role of 25(OH)D in lipid metabolism, our findings are consistent with the broader literature suggesting that adequate 25(OH)D levels are beneficial for maintaining optimal lipid profiles (17, 18, 20, 36, 37). Despite some inconsistencies in the results of previous studies, the preponderance of evidence points to a beneficial influence of adequate 25(OH)D on HDL cholesterol and a moderating effect on LDL cholesterol and triglycerides. This relationship is particularly evident in our data, which show a marked association between low 25(OH)D levels and elevated total cholesterol and triglycerides, especially in females.

Vitamin D deficiency may contribute to dyslipidemia through multiple mechanisms. It regulates genes involved in cholesterol synthesis and clearance, such as CYP7A1 and ABCA1, and modulates inflammatory pathways that play a key role in lipid metabolism (22, 23). Additionally, vitamin D deficiency is associated with insulin resistance, which can further exacerbate lipid abnormalities, particularly elevated triglycerides (26, 27). These mechanisms are supported by studies showing that vitamin D supplementation improves lipid profiles, particularly by reducing LDL-C and triglyceride levels (28, 29). Our findings align with this evidence, suggesting that maintaining adequate vitamin D levels may be crucial for optimal lipid metabolism and cardiovascular health.

Our investigation of the relationship between dyslipidemia and 25(OH)D deficiency highlights a critical public health issue with significant global prevalence, particularly in regions where vitamin D fortification is lacking, such as Europe, China, India, the Middle East, and South America (38–41). The study highlights a higher-than-expected rate of 25(OH)D insufficiency and deficiency among adults, with a notable proportion of younger individuals having lower serum 25(OH)D levels than their older counterparts. This finding calls for a reevaluation of the common perception that vitamin D deficiency primarily affects the elderly, and highlights lifestyle factors such as limited sunlight exposure due to indoor activities and increased use of sunscreen as key contributors (7, 42).

In light of these findings, our study advocates a multifaceted approach to address 25(OH)D deficiency and its association with dyslipidemia. Public health strategies should not only emphasize dietary fortification and supplementation, but also consider lifestyle modifications to enhance natural vitamin D synthesis. Furthermore, recognition of the genetic basis of this association may pave the way for personalized interventions tailored to individual risk profiles (43).

Although we observed a significant association between vitamin D deficiency and dyslipidemia, the cross-sectional design of our study limits our ability to infer causality. It is possible that 25(OH)D deficiency may contribute to alterations in lipid metabolism, or that both conditions share common underlying risk factors. The small number of participants in certain subgroups (e.g., those with 25(OH)D levels ≥75 nmol/L) may affect the generalizability of our findings. Additionally, we did not account for potential confounding factors such as dietary intake, physical activity, skin color, and sun exposure, which could independently influence vitamin D status and lipid profiles. For example, dietary intake of vitamin D-rich foods and supplements, as well as sun exposure, are known to significantly affect serum 25(OH)D levels. Similarly, physical activity may influence lipid metabolism independently of vitamin D status. Future studies should aim to collect and adjust for these variables to provide a more comprehensive understanding of the association between vitamin D deficiency and dyslipidemia. Large, longitudinal and interventional studies are essential to confirm these associations and explore the underlying mechanisms in diverse populations.

In conclusion, our study demonstrates that 25-hydroxyvitamin D deficiency is significantly associated with dyslipidemia, particularly in females, in a Chinese population. These findings suggest a potential link between vitamin D status and lipid metabolism, which warrants further investigation to clarify the underlying mechanisms and potential clinical implications.

## Data Availability

The original contributions presented in the study are included in the article/supplementary material. Further inquiries can be directed to the corresponding author/s.
